# Supplementary material for: Vegetarian Diet and Dietary Intake, Health, and Nutritional Status in Infants, Children, and Adolescents: A Systematic Review
Source: Nutrients. 2025 Jun 30;17(13):2183. doi: 10.3390/nu17132183 (PMC12251893; doi:10.3390/nu17132183)
Supplement: Supplementary file 1 [file nutrients-17-02183-s001.zip › Suppl1 Revision.pdf]

Supplemental Table S1: Assessement of risk of bias and certainity of evidence by using the NOS scale

Items / Study	Selection													Comparability		Outcome										
	Representativeness of the exposed cohort				Selection of the non exposed cohort			Ascertainment of exposure				Demonstration that outcome of interest was not present at start of study		Comparability of cohorts on the basis of the design or analysis		Assessment of outcome				Was follow-up long enough for outcomes to occur		Adequacy of follow up of cohorts				
	truly representative of the average…in the community (*)	somewhat representative of the average… in the community (*)	selected group of users eg nurses, volunteers	no description of the derivation of the cohort	drawn from the same community as the exposed cohort (*)	drawn from a different source	no description of the derivation of the non exposed cohort	secure record (eg surgical records) (*)	structured interview (*)	written self report	no description	yes (*)	no	study controls for the most important factors (*)	study controls for any additional factor (*)	independent blind assessment (*)	record linkage (*)	self report	no description	yes (*)	no	complete follow up - all subjects accounted for (*)	subjects lost to follow up unlikely to introduce bias - small number lost (*)	follow up rate < ___% (select an adequate %) and no description of those lost	no statement	total number of stars (*)
Alexy et al., 2021 (22)		*			*				*				x	*	*		*	x		N/A	N/A	N/A	N/A	N/A	N/A	6
Alexy et al., 2022 (23)		*			*				*				x	*	*			x		N/A	N/A	N/A	N/A	N/A	N/A	5
Ambroszkiewicz et al., 2017 (24)		*			*				*				x	*	*		*	x		N/A	N/A	N/A	N/A	N/A	N/A	6
Ambroszkiewicz et al. 2018 (25)		*			*				*				x	*	*		*	x		N/A	N/A	N/A	N/A	N/A	N/A	6
Ambroszkiewicz et al. 2018 (26)		*			*				*				x	*	*		*	x		N/A	N/A	N/A	N/A	N/A	N/A	6
Ambroszkiewicz et al. 2019 (27)		*			*				*				x	*	*		*	x		N/A	N/A	N/A	N/A	N/A	N/A	6
Ambroszkiewicz et al. 2021 (28)		*			*				*				x	*	*		*	x		N/A	N/A	N/A	N/A	N/A	N/A	6
Ambroszkiewicz et al. 2023 (29)		*			*				*				x	*	*		*	x		N/A	N/A	N/A	N/A	N/A	N/A	6
Desmond et al. 2021 (30)		*			*				*				x	*	*		*	x		N/A	N/A	N/A	N/A	N/A	N/A	6
Elliott et al., 2022 (31)		*			*				*			*		*	*		*	x		*				x		8
Grant et al., 2021 (32)		*			*				*				x	x	*		*	x		N/A	N/A	N/A	N/A	N/A	N/A	5
Hovinen et al., 2021 (33)		*			*				*				x	*	*		*	x		N/A	N/A	N/A	N/A	N/A	N/A	6
Nieczuja-Dwojacka et al., 2020 (34)		*			*				*				x	*	*		*	x		N/A	N/A	N/A	N/A	N/A	N/A	6
Peddie et al., 2022 (35)		*			*				*				x	*			*	x		N/A	N/A	N/A	N/A	N/A	N/A	5
Rowicka et al., 2023 (36)		*			*				*				x	*	*		*	x		N/A	N/A	N/A	N/A	N/A	N/A	6
Segovia-Siapco et al., 2019 (37)			x		*				*				x	*			*	x		N/A	N/A	N/A	N/A	N/A	N/A	4
Svetnicka et al., 2022 (38)		*			*				*				x	*	*		*	x		N/A	N/A	N/A	N/A	N/A	N/A	6
Weder et al., 2019 (39)		*			*				*				x	*	*		*	x		N/A	N/A	N/A	N/A	N/A	N/A	6
Weder et al., 2022 (40)		*			*				*				x	*	*		*	x		N/A	N/A	N/A	N/A	N/A	N/A	6
Weder et al., 2023 (41)		*			*				*				x	*	*		*	x		N/A	N/A	N/A	N/A	N/A	N/A	6

Newcastle-Ottawa Scale Wells G, Shea B, O'Connell D, Peterson J, Welch V, Losos M, Tugwell P. The Newcastle-Ottawa Scale (NOS) for assessing the quality of nonrandomised studies in meta-analyses. Internet: https://www.ohri.ca/programs/clinical_epidemiology/oxford.asp
