# Supplementary material for: Vegetarian Diet and Dietary Intake, Health, and Nutritional Status in Infants, Children, and Adolescents: A Systematic Review
Source: Nutrients. 2025 Jun 30;17(13):2183. doi: 10.3390/nu17132183 (PMC12251893; doi:10.3390/nu17132183)
Supplement: Supplementary file 1 [file nutrients-17-02183-s001.zip › Supplemental tables review revisionDR.pdf]

Supplemental:

**Supplemental Table S2: Anthropometric data of children with VG and OM diet. Data are taken from the references given.**

| | | BMI | | | | Height | | | | Weight | | | |
|---|---|---|---|---|---|---|---|---|---|---|---|---|---|
| **Reference** | **Age group [y]** | **Unit** | **VG** | **OM** | **p-value** | **Height** | **VG** | **OM** | **p-value** | **Unit** | **VG** | **OM** | **p-value** |
| Alexy et al., 2021 (22) | 5-19 | SDS | -0.3 | -0.3 | N/A | cm | 154 | 156 | N/A | kg | 45 | 46 | N/A |
| Ambroszkiewicz et al., 2017 (24) | 4-9 | $kg/m^2$ | 15.24 | 14.86 | 0.159 | cm | 122 | 118 | 0.129 | kg | 23.4 | 20.9 | 0.088 |
| Ambroszkiewicz et al., 2018 (25) | 5-10 | $kg/m^2$ | 15.4 | 15.5 | 0.76 | cm | 119.2 | 122.3 | 0.114 | kg | 22.16 | 23.26 | 0.275 |
| | | z-score | -0.35 | -0.30 | 0.674 | | | | | | | | |
| Ambroszkiewicz et al., 2018 (26) | 5-10 | $kg/m^2$ | 15.3 | 14.8 | 0.078 | cm | 119.9 | 120.2 | 0.89 | kg | 22.1 | 22.1 | 0.975 |
| Ambroszkiewicz et al., 2018 (27) | 5-10 | $kg/m^2$ | 15.4 | 14.9 | 0.27 | cm | 122.2 | 123.2 | 0.414 | kg | 21.0 | 21.4 | 0.771 |
| Ambroszkiewicz et al., 2021 (28) | 5-9 | $kg/m^2$ | 14.76 | 14.66 | 0.07 | cm | 117.39 | 118.58 | 0.544 | kg | 20.7 | 21.22 | 0.723 |
| Ambroszkiewic et al., 2023 (29) | 5-9 | $kg/m^2$ | 14.9 | 15.1 | 0.573 | N/A | N/A | N/A | N/A | N/A | N/A | N/A | N/A |
| Desmond et al., 2021 (30) | 5-10 | z-score | -0.27 | -0.03 | >0.05 | z-score | 0.18 | 0.62 | **< 0.05** | z-score | -0.07 | 0.31 | N/A |
| Elliott et al., 2022 (31) | 0.5-8 [1)] | z-score | -0.11 | 0.04 | 0.79 | z-score | 0.18 | 0.32 | **0.02** | N/A | N/A | N/A | N/A |
| Grant et al., 2021 (32) | 14-17 [1)] | $kg/m^2$ | 20.8 | 21.5 | 0.159 | N/A | N/A | N/A | N/A | N/A | N/A | N/A | N/A |
| Hovinen et al., 2021 (33) | 1-7 | SDS | 0.54 | 0.27 | N/A | z-score | -0.88 | -0.44 | N/A | N/A | N/A | N/A | N/A |
| Nieczuja-Dwojacka et al., 2020 (34) | 3-15 [1)] | $kg/m^2$ | N/A | N/A | **< 0.01** | cm | N/A | N/A | **< 0.05** | N/A | N/A | N/A | N/A |
| Peddie et al., 2022 (35) | 15-18 [1),2)] | z-score | 0.25 | 0.76 | **0.003** | cm | 166 | 166 | 0.912 | N/A | N/A | N/A | N/A |
| Rowicka et al., 2023 (36) | 2-10 | $kg/m^2$ | 15.40 | 15.50 | 0.056 | cm | 121.5 | 121 | 0.91 | kg | 22.7 | 22.1 | 0.371 |
| | | z-score | -0.18 | 0.19 | 0.06 | | | | | | | | |
| Segovia-Siapco et al 2019 (37) | 12-18 [1)] | z-score | 0.14 | 0.42 | **0.005** | N/A | N/A | N/A | N/A | N/A | N/A | N/A | N/A |
| Svetnicka et al 2022 (38) | 0-18 | Percen-tile | 42.00 | 40.00 | N/A | Percen-tile | 48.00 | 45.00 | 0.883 | Percen-tile | 47.00 | 44.00 | 0.386 |
| Weder et al. 2019 (39), Weder et al, 2022 (40), Weder et al 2023 (41) | 1-3 | N/A | N/A | N/A | N/A | cm | 86.6 | 88.2 | N/A | kg | 12.1 | 12.7 | N/A |

[1)] VEs are included in VG group; [2)]only females

N/A: Not available; Significant p-values are shown in bold. Only p-values of unadjusted data sets were considered.

**Supplemental Table S3: Supplemental data for Fig.2 (A-C; age group:1-18 y).**

| | BMI [kg/m2] | | Height [cm] | | Weight [kg] | |
|---|---|---|---|---|---|---|
| | **VG** | **OM** | **VG** | **OM** | **VG** | **OM** |
| Number of values | 8 | 8 | 8 | 8 | 8 | 8 |
| Minimum | 14.76 | 14.66 | 86.60 | 88.20 | 12.10 | 12.70 |
| 25% Percentile | 14.99 | 14.82 | 118.3 | 118.3 | 20.78 | 20.98 |
| Median | 15.35 | 15.00 | 121.5 | 121.0 | 22.13 | 21.75 |
| 75% Percentile | 15.40 | 15.50 | 138.1 | 139.6 | 23.23 | 22.97 |
| Maximum | 20.80 | 21.50 | 166.0 | 166.0 | 45.00 | 46.00 |
| Mean | 15.90 | 15.85 | 125.4 | 125.9 | 23.65 | 23.71 |
| Std. Deviation | 1.995 | 2.303 | 22.75 | 22.72 | 9.339 | 9.583 |
| Std. Error | 0.7053 | 0.8143 | 7.584 | 7.572 | 3.302 | 3.388 |
| Lower 95% CI of mean | 14.23 | 13.93 | 107.9 | 108.5 | 15.84 | 15.70 |
| Upper 95% CI of mean | 17.57 | 17.78 | 142.9 | 143.4 | 31.45 | 31.72 |
| **P value** | **0.9655** | | **0.9618** | | **0.9892** | |

**Supplemental Table S4: Supplemental data for Fig.2 (D-F; age group:2-10 y).**

| | BMI [kg/m2] | | Height [cm] | | Weight [kg] | |
|---|---|---|---|---|---|---|
| | VG | OM | VG | OM | VG | OM |
| Number of values | 6 | 6 | 6 | 6 | 6 | 6 |
| Minimum | 20.70 | 20.90 | 117.4 | 118.0 | 20.7 | 20.9 |
| 25% Percentile | 20.93 | 21.14 | 118.7 | 118.4 | 20.93 | 21.14 |
| Median | 22.13 | 21.75 | 120.7 | 120.6 | 22.13 | 21.75 |
| 75% Percentile | 22.88 | 22.39 | 122.1 | 122.5 | 22.88 | 22.39 |
| Maximum | 23.40 | 23.26 | 122.2 | 123.2 | 23.40 | 23.26 |
| Mean | 22.01 | 21.83 | 120.4 | 120.5 | 22.01 | 21.83 |
| Std. Deviation | 1.017 | 0.8500 | 1.884 | 2.039 | 1.017 | 0.850 |
| Std. Error | 0.4153 | 0.3470 | 0.7693 | 0.8325 | 0.4153 | 0.3470 |
| Lower 95% CI of mean | 20.94 | 20.94 | 118.4 | 118.4 | 20.94 | 20.94 |
| Upper 95% CI of mean | 23.08 | 22.72 | 122.3 | 122.7 | 23.07 | 22.72 |
| **P value** | **0.7463** | | **0.8759** | | **0.7463** | |

**Supplemental Table S5: Intake of selected nutrients of children with VG and OM diet. Data are taken from the references given.**

| | | Fibre | | | | Carbohydrate | | | | Protein | | | | Fat | | | |
|---|---|---|---|---|---|---|---|---|---|---|---|---|---|---|---|---|---|
| **Reference** | **Age group [y]** | **Unit** | **VG** | **OM** | **p-value** | **Unit** | **VG** | **OM** | **p-value** | **Unit** | **VG** | **OM** | **p-value** | **Unit** | **VG** | **OM** | **p-value** |
| Alexy et al., 2021 (22) | 5-19 | g/ 1000 kcal | 14.7 | 12.0 | **0.0006** | N/A | N/A | N/A | N/A | g/kg BW /day | 1.1 | 1.4 | N/A | N/A | N/A | N/A | N/A |
| Ambroszkiewicz et al., 2017 (24) | 4-9 | N/A | N/A | N/A | N/A | g/day | 223.9 | 208.8 | 0.495 | g/day | 42.5 | 51.2 | 0.057 | g/day | 53.2 | 60.1 | 0.329 |
| Ambroszkiewicz et al., 2018 (26) | 5-10 | g/day | 19.3 | 15.6 | **0.01** | g/day | 218.5 | 213.9 | 0.713 | g/day | 41.7 | 53.2 | **<0.001** | g/day | 52.8 | 58.5 | 0.234 |
| Ambroszkiewicz et al., 2018 (27) | 5-10 | g/day | 20.4 | 14.2 | **0.015** | N/A | N/A | N/A | N/A | g/day | 37.7 | 54.8 | **0.002** | N/A | N/A | N/A | N/A |
| Ambroszkiewicz et al., 2021 (28) | 5-9 | g/day | 19 | 16 | 0.063 | N/A | N/A | N/A | N/A | N/A | N/A | N/A | N/A | N/A | N/A | N/A | N/A |
| Ambroszkiewic et al., 2023 (29) | 5-9 | g/day | 16.7 | 14.8 | **0.0034** | N/A | N/A | N/A | N/A | g/day | 35.5 | 54.9 | **0.0009** | N/A | N/A | N/A | N/A |
| Desmond et al., 2021 (30) | 5-10 | g/day | 24.7 | 14.9 | N/A | g/day | 243.0 | 216.0 | N/A | g/day | 45.1 | 57.3 | N/A | g/day | 53.6 | 65.7 | N/A |
| Hovinen et al., 2021 (33) | 1-7 | g/day | 24 | 15 | N/A | N/A | N/A | N/A | N/A | N/A | N/A | N/A | N/A | N/A | N/A | N/A | N/A |
| Peddie et al., 2022 (35) | 15-18 [1)] | g/day | 27.4 | 23.5 | **0.019** | g/day | 220.0 | 225.0 | 0.589 | g/day | 61.3 | 74.6 | **<0.001** | g/day | 76.0 | 79.7 | 0.366 |
| Rowicka et al., 2023 (36) | 2-10 | g/day | 18.5 | 14.2 | **0.012** | N/A | N/A | N/A | N/A | N/A | N/A | N/A | N/A | N/A | N/A | N/A | N/A |
| Segovia-Siapco et al., 2019 (37) | 12-18 [1)] | g/day | 29.84 | 21.9 | **<0.0001** | g/day | 275.1 | 248.6 | **<0.0001** | g/day | 77.5 | 79.8 | 0.124 | g/day | 70.9 | 77.1 | **<0.0001** |
| Weder et al., 2019 (39), 2022 (40) | 1-3 | g/ 1000 kcal | 16.1 | 13.4 | N/A | N/A | N/A | N/A | N/A | g/kg BW /day | 2.3 | 2.5 | **p<0.001** | N/A | N/A | N/A | N/A |

[1)] VEs are included in VG group.; N/A: Not available; Significant p-values are shown in bold.; N/A: Not available; Significant p-values are shown in bold. Only p-values of unadjusted data sets were considered.

**Supplemental Table S6: Supplemental data for Fig.4(A-D; age group:1-18 y).**

| | **Fiber [g/day]** | | **Carbohydrates [g/day]** | | **Protein [g/day]** | | **Fat [g/day]** | |
|---|---|---|---|---|---|---|---|---|
| | **VG** | **OM** | **VG** | **OM** | **VG** | **OM** | **VG** | **OM** |
| Number of values | 9 | 9 | 5 | 5 | 7 | 7 | 5 | 5 |
| Minimum | 16.70 | 14.20 | 218.5 | 208.8 | 35.50 | 51.20 | 52.80 | 58.50 |
| 25% Percentile | 18.75 | 14.50 | 219.3 | 211.4 | 37.70 | 53.20 | 53.00 | 59.30 |
| Median | 20.40 | 15.00 | 223.9 | 216.0 | 42.50 | 54.90 | 53.60 | 65.70 |
| 75% Percentile | 26.05 | 18.94 | 259.0 | 236.8 | 61.30 | 74.60 | 73.48 | 78.40 |
| Maximum | 29.84 | 23.50 | 275.1 | 248.6 | 77.48 | 79.84 | 76.00 | 79.70 |
| Mean | 22.20 | 16.67 | 236.1 | 222.5 | 48.75 | 60.83 | 61.31 | 68.22 |
| Std. Deviation | 4.482 | 3.480 | 23.89 | 15.76 | 15.18 | 11.45 | 11.25 | 9.711 |
| Std. Error | 1.494 | 1.160 | 10.68 | 7.048 | 5.738 | 4.326 | 5.032 | 4.343 |
| Lower 95% CI of mean | 18.76 | 14.00 | 206.4 | 202.9 | 34.71 | 50.25 | 47.34 | 56.16 |
| Upper 95% CI of mean | 25.65 | 19.35 | 265.8 | 242.0 | 62.79 | 71.42 | 75.28 | 80.28 |
| P value | **0.0099** | | 0.3182 | | 0.1186 | | 0.3291 | |

**Supplemental Table S7: Supplemental data for Fig.4 (E-H; age group:2-10 y).**

| | Fiber [g/day] | | Carbohydrates [g/day] | | Protein [g/day] | | Fat [g/day] | |
|---|---|---|---|---|---|---|---|---|
| | VG | OM | VG | OM | VG | OM | VG | OM |
| Number of values | 6 | 6 | 3 | 3 | 5 | 5 | 3 | 3 |
| Minimum | 16.70 | 14.20 | 218.5 | 208.8 | 35.50 | 51.20 | 52.80 | 58.50 |
| 25% Percentile | 18.05 | 14.20 | 218.5 | 208.8 | 36.60 | 52.20 | 52.80 | 58.50 |
| Median | 19.15 | 14.85 | 223.9 | 213.9 | 41.70 | 54.80 | 53.20 | 60.10 |
| 75% Percentile | 21.48 | 15.70 | 243.0 | 216.0 | 43.80 | 56.10 | 53.60 | 65.70 |
| Maximum | 24.70 | 16.00 | 243.0 | 216.0 | 45.10 | 57.30 | 53.60 | 65.70 |
| Mean | 19.77 | 14.95 | 228.5 | 212.9 | 40.50 | 54.28 | 53.20 | 61.43 |
| Std. Deviation | 2.704 | 0.7314 | 12.87 | 3.703 | 3.855 | 2.260 | 0.4000 | 3.781 |
| Std. Error | 1.104 | 0.2986 | 7.432 | 2.138 | 1.724 | 1.011 | 0.2309 | 2.183 |
| Lower 95% CI of mean | 17.65 | 14.34 | 196.5 | 203.7 | 35.71 | 51.47 | 52.21 | 52.04 |
| Upper 95% CI of mean | 23.09 | 15.57 | 260.4 | 222.1 | 45.29 | 57.09 | 54.19 | 70.82 |
| P value | 0.005 | | 0.1144 | | 0.0001 | | 0.0199 | |

**Supplemental Table S8: Intake of energy of children with VG and OM diet. Data are taken from the references given.**

| | | Total Energy | | | | Energy from carbohydrates | | | | Energy from protein | | | | Energy from fat | | | |
|---|---|---|---|---|---|---|---|---|---|---|---|---|---|---|---|---|---|
| **Reference** | **Age group [y]** | **Unit** | **VG** | **OM** | **p-value** | **Unit** | **VG** | **OM** | **p-value** | **Unit** | **VG** | **OM** | **p-value** | **Unit** | **VG** | **OM** | **p-value** |
| Alexy et al., 2021 (22) | 5-19 | kcal/day | 1708.0 | 1737.0 | N/A | % | 54.7 | 49.1 | N/A | N/A | N/A | N/A | N/A | % | 32.3 | 36.4 | N/A |
| Ambroszkiewicz et al., 2017 (24) | 4-9 | kcal/day | 1443.4 | 1551.5 | 0.391 | % | 57.4 | 51.5 | **0.001** | % | 12.4 | 13.7 | **0.03** | % | 30.2 | 34.8 | 0.1 |
| Ambroszkiewicz et al., 2018 (25) | 5-10 | N/A | N/A | N/A | N/A | % | 57.1 | 52.3 | **0.004** | % | 11.4 | 15.6 | **<0.001** | % | 31.5 | 32.8 | 0.318 |
| Ambroszkiewicz et al., 2018 (26) | 5-10 | kcal/day | 1445.0 | 1539.0 | 0.303 | N/A | N/A | N/A | N/A | % | 11.7 | 14.1 | **<0.001** | % | 31.6 | 32.8 | 0.286 |
| Ambroszkiewicz et al., 2018 (27) | 5-10 | kcal/day | 1376.0 | 1615.0 | 0.116 | % | 58.2 | 52.0 | **0.001** | % | 11.0 | 14.1 | **0.001** | % | 30.8 | 33.9 | **0.043** |
| Ambroszkiewicz et al., 2021 (28) | 5-9 | kcal/day | 1404.6 | 1590.6 | 0.470 | % | 56.7 | 52.9 | **0.002** | % | 11.5 | 13.6 | **<0.001** | % | 31.1 | 33.3 | 0.166 |
| Ambroszkiewicz et al., 2023 (29) | 5-9 | kcal/day | 1396.0 | 1476.0 | 0.408 | % | 56.7 | 52.2 | **0.012** | % | 12.8 | 15.8 | **0.0002** | % | 30.5 | 32 | 0.379 |
| Desmond et al., (2021) (30) | 5-10 | kcal/day | 1564.0 | 1637.0 | N/A | N/A | N/A | N/A | N/A | N/A | N/A | N/A | N/A | N/A | N/A | N/A | N/A |
| Hovinen et al., 2021 (33) | 1-7 | kcal/day | 1232.4 | 1225.2 | N/A | % | 45.9 | 48.2 | N/A | % | 14.5 | 16.4 | N/A | % | 34.7 | 32.5 | N/A |
| Peddie et al., 2022 (35) | 15-18 [1)] | kcal/day | 1795.8 | 1916.2 | 0.128 | % | 48.7 | 46.9 | 0.082 | % | 13.6 | 15.6 | **<0.001** | % | 38.2 | 37.3 | 0.369 |
| Rowicka et al., 2023 (36) | 2-10 | kcal/day | 1499.3 | 1558.4 | 0.330 | % | 56.7 | 52.9 | **0.005** | % | 11.6 | 13.2 | **0.01** | % | 28.9 | 31.3 | 0.079 |
| Segovia-Siapco et al., 2019 (37) | 12-18 [1)] | kcal/day | 2010.2 | 1990.2 | 0.277 | N/A | N/A | N/A | N/A | N/A | N/A | N/A | N/A | N/A | N/A | N/A | N/A |
| Weder et al., 2019 (39), 2022 (40) | 1-3 | kcal/day | 956.0 | 974.0 | n.s. | % | 53.6 | 53.1 | n.s. | % | 11.1 | 13.2 | **<0.001** | % | 33.7 | 32.6 | n.s. |

[1)] VEs are included in VG group.; N/A: Not available; Significant p-values are shown in bold. Only p-values of unadjusted data sets were considered.

**Supplemental Table S9: Supplemental data for Fig.3 (A-D; age group:1-18 y).**

| | Total energy intake [kcal/day] | | Energy from carbohydrate [%] | | Energy from protein [%] | | Energy from fat [%] | |
|---|---|---|---|---|---|---|---|---|
| | **OM** | **VG** | **OM** | **VG** | **OM** | **OM** | **VG** | **OM** |
| Number of values | 12 | 12 | 10 | 10 | 10 | 10 | 11 | 11 |
| Minimum | 956.0 | 974.0 | 45.90 | 46.90 | 11.00 | 13.20 | 28.90 | 31.30 |
| 25% Percentile | 1381 | 1492 | 52.38 | 48.88 | 11.33 | 13.50 | 30.50 | 32.50 |
| Median | 1444 | 1574 | 56.70 | 52.10 | 11.65 | 14.10 | 31.50 | 32.80 |
| 75% Percentile | 1672 | 1712 | 57.18 | 52.90 | 13.00 | 15.65 | 33.70 | 34.80 |
| Maximum | 2010 | 1990 | 58.20 | 53.10 | 14.50 | 16.40 | 38.20 | 37.30 |
| Mean | 1486 | 1568 | 54.57 | 51.11 | 12.16 | 14.53 | 32.14 | 33.61 |
| Std. Deviation | 270.0 | 272.3 | 4.107 | 2.215 | 1.156 | 1.195 | 2.574 | 1.857 |
| Std. Error | 77.94 | 78.60 | 1.299 | 0.7004 | 0.3655 | 0.3780 | 0.7762 | 0.5599 |
| Lower 95% CI of mean | 1314 | 1395 | 51.63 | 49.53 | 11.33 | 13.67 | 30.41 | 32.36 |
| Upper 95% CI of mean | 1657 | 1741 | 57.51 | 52.69 | 12.99 | 15.39 | 33.87 | 34.86 |
| P value | 0.4687 | | **0.0307** | | **0.0003** | | 0.1395 | |

**Supplemental Table S10: Supplemental data for Fig.3 (E-H; age group:2-10 y).**

| | Total energy intake [kcal/day] | | Energy from carbohydrate [%] | | Energy from protein [%] | | Energy from fat [%] | |
|---|---|---|---|---|---|---|---|---|
| | **OM** | **VG** | **OM** | **VG** | **OM** | **OM** | **VG** | **OM** |
| Number of values | 7 | 7 | 6 | 6 | 7 | 7 | 7 | 7 |
| Minimum | 1376 | 1476 | 56.70 | 51.50 | 11.00 | 13.20 | 28.90 | 31.30 |
| 25% Percentile | 1396 | 1539 | 56.70 | 51.88 | 11.40 | 13.60 | 30.20 | 32.00 |
| Median | 1443 | 1558 | 56.90 | 52.25 | 11.60 | 14.10 | 30.80 | 32.80 |
| 75% Percentile | 1499 | 1615 | 57.60 | 52.90 | 12.40 | 15.60 | 31.50 | 33.90 |
| Maximum | 1564 | 1637 | 58.20 | 52.90 | 12.80 | 15.80 | 31.60 | 34.80 |
| Mean | 1447 | 1567 | 57.13 | 52.30 | 11.77 | 14.30 | 30.66 | 32.99 |
| Std. Deviation | 65.68 | 53.45 | 0.5955 | 0.5404 | 0.6184 | 1.007 | 0.9253 | 1.163 |
| Std. Error | 24.82 | 20.20 | 0.2431 | 0.2206 | 0.2337 | 0.3805 | 0.3497 | 0.4394 |
| Lower 95% CI of mean | 1386 | 1517 | 56.51 | 51.73 | 11.20 | 13.37 | 29.80 | 31.91 |
| Upper 95% CI of mean | 1508 | 1616 | 57.76 | 52.87 | 12.34 | 15.23 | 31.51 | 34.06 |
| P value | **0.0028** | | **<0.0001** | | **0.0001** | | **0.0014** | |

**Supplemental Table S11: Intake of selected minerals of children with VG and OM diet. Data are taken from the references given.**

| | | | Calcium | | | | Magnesium | | | | Iron | | | |
|---|---|---|---|---|---|---|---|---|---|---|---|---|---|---|
| **Reference** | **Age group [y]** | **Supple-ments** | **Unit** | **VG** | **OM** | **p-value** | **Unit** | **VG** | **OM** | **p-value** | **Unit** | **VG** | **OM** | **p-value** |
| Alexy et al.. 2021 (22) | 5-19 | - | mg/ 1000 kcal | 390 | 400 | N/A | mg/ 1000 kcal | 176 | 153 | N/A | mg/ 1000 kcal | 6.8 | 5.7 | N/A |
| Ambroszkiewicz et al.. 2017 (24) | 4-9 | N/A | N/A | N/A | N/A | N/A | N/A | N/A | N/A | N/A | mg/day | 9.5 | 8.9 | 0.150 |
| Ambroszkiewicz et al.. 2018 (25) | 5-10 | N/A | mg/day | 548 | 596 | 0.410 | N/A | N/A | N/A | N/A | N/A | N/A | N/A | N/A |
| Ambroszkiewicz et al.. 2018 (27) | 5-10 | N/A | mg/day | 460 | 529 | 0.285 | mg/day | 248 | 193 | 0.054 | N/A | N/A | N/A | N/A |
| Ambroszkiewicz et al.. 2021 (28) | 5-9 | N/A | mg/day | 513.3 | 604 | 0.063 | mg/day | 223.5 | 216.7 | 0.633 | N/A | N/A | N/A | N/A |
| Ambroszkiewicz et al 2023 (29) | 5-9 | N/A | mg/day | 504.7 | 616.5 | **0.015** | mg/day | 229.8 | 217.4 | 0.661 | N/A | N/A | N/A | N/A |
| Desmond et al.. 2021 (30) | 5-10 | + | mg/day | 607.0 | 547 | N/A | mg/day | 301 | 223 | N/A | mg/day | 11.5 | 8.6 | N/A |
| Hovinen et al.. 2021 (33) | 1-7 | + | mg/day | 893 | 874 | N/A | mg/day | 301 | 246 | N/A | mg/day | 10.8 | 7.3 | N/A |
| | | - | mg/day | 893 | 880 | N/A | mg/day | 301 | 246 | N/A | mg/day | 10.8 | 7.4 | N/A |
| Segovia-Siapco et al.. 2019 (37) | 12-18 [1)] | + | mg/day | 1221.7 | 1091.2 | **<0.0001** | mg/day | 386.4 | 314.5 | **<0.0001** | mg/day | 20.2 | 16.6 | **<0.0001** |
| Weder et al.. 2022 (40) | 1-3 | + | mg/day | 392 | 452 | N/A | mg/day | 189 | 164 | N/A | mg/day | 7.3 | 6 | N/A |
| | | - | mg/day | 399 | 445 | N/A | mg/day | 188 | 164 | N/A | mg/day | 7.3 | 6 | N/A |

[1)] VEs are included in VG group; N/A: Not available; Significant p-values are shown in bold. Only p-values of unadjusted data sets were considered.

**Supplemental Table S12: Supplemental data for Fig.5 (A-C; age group:1-18 y).**

| | Calcium [mg/day] | | Magnesium [mg/day] | | Iron [mg/day] | |
|---|---|---|---|---|---|---|
| | **VG** | **OM** | **VG** | **OM** | **VG** | **OM** |
| Number of values | 8 | 8 | 7 | 7 | 5 | 5 |
| Minimum | 392.0 | 452.0 | 189.0 | 164.0 | 7.300 | 6.000 |
| 25% Percentile | 471.2 | 533.5 | 223.5 | 193.0 | 8.400 | 6.650 |
| Median | 530.7 | 600.0 | 248.0 | 217.4 | 10.80 | 8.600 |
| 75% Percentile | 821.5 | 809.6 | 301.0 | 246.0 | 15.85 | 12.74 |
| Maximum | 1222 | 1091 | 386.5 | 314.5 | 20.19 | 16.58 |
| Mean | 642.5 | 663.7 | 268.4 | 224.9 | 11.86 | 9.476 |
| Std. Deviation | 277.8 | 211.8 | 66.19 | 47.13 | 4.925 | 4.135 |
| Std. Error | 98.21 | 74.87 | 25.02 | 17.81 | 2.202 | 1.849 |
| Lower 95% CI of mean | 410.2 | 486.7 | 410.2 | 486.7 | 5.743 | 4.342 |
| Upper 95% CI of mean | 874.7 | 840.7 | 874.7 | 840.7 | 17.97 | 14.61 |
| P value | 0.8659 | | 0.1826 | | 0.4315 | |

**Supplemental Table S13: Supplemental data for Fig.5 (D-F; age group:2-10 y).**

| | Calcium [mg/day] | | Magnesium [mg/day] | | Iron [mg/day] | |
|---|---|---|---|---|---|---|
| | **VG** | **OM** | **VG** | **OM** | **VG** | **OM** |
| Number of values | 5 | 5 | 4 | 4 | 2 | 2 |
| Minimum | 460.0 | 529.0 | 223.5 | 193.0 | 9.500 | 8.600 |
| 25% Percentile | 482.4 | 538.0 | 225.1 | 198.9 | 9.500 | 8.600 |
| Median | 513.3 | 596.0 | 238.9 | 217.1 | 10.50 | 8.750 |
| 75% Percentile | 577.5 | 610.3 | 287.8 | 221.6 | 11.50 | 8.900 |
| Maximum | 607.0 | 616.5 | 301.0 | 223.0 | 11.50 | 8.900 |
| Mean | 526.6 | 578.5 | 250.6 | 212.5 | 10.50 | 8.750 |
| Std. Deviation | 54.80 | 38.22 | 35.19 | 13.32 | 1.414 | 0.2121 |
| Std. Error | 24.51 | 17.09 | 17.59 | 6.659 | 1.000 | 0.1500 |
| Lower 95% CI of mean | 458.6 | 531.0 | 194.6 | 191.3 | -2.206 | 6.844 |
| Upper 95% CI of mean | 594.6 | 626.0 | 306.6 | 233.7 | 23.21 | 10.66 |
| P value | 0.1206 | | 0.1206 | | 0.2257 | |

**Supplemental Table S14: Intake of selected vitamins of children with VG and OM diet. Data are taken from the references given.**

| | | | Vitamin B12 | | | | Vitamin C | | | | Vitamin D | | | | Vitamin E | | | |
|---|---|---|---|---|---|---|---|---|---|---|---|---|---|---|---|---|---|---|
| **Reference** | **Age group [y]** | **Supple-ments** | **Unit** | **VG** | **OM** | **p-value** | **Unit** | **VG** | **OM** | **p-value** | **Unit** | **VG** | **OM** | **p-value** | **Unit** | **VG** | **OM** | **p-value** |
| Alexy et al.. 2021 (22) | 5-19 | - | µg/ 1000 kcal | 0.6 | 1.6 | N/A | mg/ 1000 kcal | 45 | 44 | N/A | N/A | N/A | N/A | N/A | mg/ 1000 kcal | 7.2 | 6 | N/A |
| Ambroszkiewicz et al..2017 (24) | 5-9 | N/A | µg/d | 1.7 | 1.8 | 0.134 | mg/d | 85.0 | 60.0 | **0.015** | N/A | N/A | N/A | N/A | N/A | N/A | N/A | N/A |
| Ambroszkiewicz et al. 2018 (25) | 5-10 | N/A | µg/d | 1.4 | 2.5 | **<0.001** | N/A | N/A | N/A | N/A | µg/d | 1.7 | 2.0 | 0.575 | N/A | N/A | N/A | N/A |
| Ambroszkiewicz et al. 2018 (27) | 5-10 | N/A | N/A | N/A | N/A | N/A | N/A | N/A | N/A | N/A | µg/d | 2.0 | 2.1 | 0.946 | N/A | N/A | N/A | N/A |
| Ambroszkiewicz et al..2021 (28) | 5-10 | N/A | µg/d | 1.44 | 2.31 | **<0.001** | mg/d | 79.8 | 68.7 | **0.019** | µg/d | 1.7 | 2.1 | 0.058 | mg/d | 9.5 | 8.04 | 0.315 |
| Ambroszkiewicz et al.. 2023 (29) | 5-10 | N/A | N/A | N/A | N/A | N/A | N/A | N/A | N/A | N/A | µg/d | 1.7 | 2.3 | **0.0116** | N/A | N/A | N/A | N/A |
| Desmond et al.. 2021 (30) | 5-10 | + | µg/d | 2.4 | 2.7 | N/A | mg/d | 135.0 | 105.0 | N/A | µg/d | 1.6 | 2.7 | N/A | N/A | N/A | N/A | N/A |
| | | - | µg/d | 1.7 | 2.7 | N/A | N/A | N/A | N/A | N/A | µg/d | 1.0 | 2.0 | N/A | N/A | N/A | N/A | N/A |
| Hovinen et al.. 2021 (33) | 1-7 | + | µg/d | 3.6 | 3.8 | N/A | mg/d | 86.0 | 80.0 | N/A | µg/d | 17.6 | 18.2 | N/A | mg/d | 10.1 | 6.2 | 0.05 |
| | | - | µg/d | 2.7 | 3.5 | N/A | mg/d | 63.3 | 68.8 | N/A | µg/d | 7.2 | 7.3 | N/A | mg/d | 7.8 | 5.6 | 0.004 |
| Rowicka et al.. 2023 (36) | 2-10 | N/A | µg/d | 1.2 | 2 | **<0.001** | mg/d | 106.7 | 87.7 | 0.604 | µg/d | 1.4 | 1.4 | 0.781 | mg/d | 9.6 | 9.1 | 0.277 |
| Segovia-Siapco et al.. 2019 (37) | 12-18 [1)] | + | µg/d | 5.8 | 5.7 | 0.539 | mg/d | 162.4 | 142.7 | **0.029** | µg/d | 4.2 | 4.7 | 0.067 | mg/d | 9.84 | 8.46 | **<0.0001** |
| Weder et al.. 2022 (40) | 1-3 | + | µg/d | 1.3 | 1.6 | N/A | mg/d | 56.0 | 47.0 | N/A | µg/d | 2.1 | 1.7 | N/A | mg/d | 7.4 | 5.1 | N/A |
| | | - | µg/d | 0.6 | 1.5 | N/A | mg/d | 54.0 | 45.0 | N/A | µg/d | 0.8 | 0.8 | N/A | mg/d | 7.4 | 5.1 | N/A |

[1)] VEs are included in VG group; N/A: Not available; Significant p-values are shown in bold. Only p-values of unadjusted data sets were considered.

**Supplemental Table S15: Supplemental data for Fig.6 (A-D; age group:1-18 y).**

| | Vitamin $B_{12}$ [µg/day] | | Vitamin C [mg/day] | | Vitamin D [µg/day] | | Vitamin E [mg/day] | |
|---|---|---|---|---|---|---|---|---|
| | VG | OM | VG | VG | OM | OM | VG | OM |
| Number of values | 8 | 8 | 7 | 7 | 9 | 9 | 5 | 5 |
| Minimum | 1.200 | 1.600 | 56.00 | 47.00 | 1.400 | 1.400 | 7.400 | 5.100 |
| 25% Percentile | 1.315 | 1.850 | 79.80 | 60.00 | 1.855 | 1.650 | 8.450 | 5.650 |
| Median | 1.570 | 2.410 | 86.00 | 80.00 | 2.120 | 1.740 | 9.600 | 8.040 |
| 75% Percentile | 3.300 | 3.525 | 135.0 | 105.0 | 3.695 | 3.140 | 9.970 | 8.780 |
| Maximum | 5.850 | 5.690 | 162.4 | 142.7 | 18.20 | 17.60 | 10.10 | 9.100 |
| Mean | 2.356 | 2.801 | 101.6 | 84.45 | 4.136 | 3.782 | 9.288 | 7.380 |
| Std. Deviation | 1.624 | 1.350 | 36.31 | 31.88 | 5.358 | 5.247 | 1.081 | 1.670 |
| Std. Error | 0.5743 | 0.4775 | 13.72 | 12.05 | 1.786 | 1.749 | 0.4833 | 0.7467 |
| Lower 95% CI of mean | 0.9982 | 1.672 | 67.97 | 54.96 | 108.5 | 107.9 | 7.946 | 5.307 |
| Upper 95% CI of mean | 3.714 | 3.930 | 135.1 | 113.9 | 143.4 | 142.9 | 10.63 | 9.453 |
| P value | 0.5608 | | 0.3674 | | 0.8849 | | 0.0643 | |

**Supplemental Table S16: Supplemental data for Fig.6 (E-H; age group:2-10 y).**

| | Vitamin $B_{12}$ [µg/day] | | Vitamin C [mg/day] | | Vitamin D [µg/day] | | Vitamin E [mg/day] | |
|---|---|---|---|---|---|---|---|---|
| | VG | OM | VG | VG | OM | OM | VG | OM |
| Number of values | 5 | 5 | 4 | 4 | 6 | 6 | 2 | 2 |
| Minimum | 1.200 | 1.800 | 79.80 | 60.00 | 1.400 | 1.400 | 9.500 | 8.040 |
| 25% Percentile | 1.280 | 1.900 | 81.10 | 62.18 | 1.550 | 1.858 | 9.500 | 8.040 |
| Median | 1.440 | 2.310 | 95.85 | 78.20 | 1.715 | 2.110 | 9.550 | 8.570 |
| 75% Percentile | 2.050 | 2.605 | 127.9 | 100.7 | 1.803 | 2.400 | 9.600 | 9.100 |
| Maximum | 2.400 | 2.700 | 135.0 | 105.0 | 1.990 | 2.700 | 9.600 | 9.100 |
| Mean | 1.620 | 2.264 | 101.6 | 80.35 | 1.693 | 2.105 | 9.550 | 8.570 |
| Std. Deviation | 0.4720 | 0.3666 | 25.12 | 20.10 | 0.1930 | 0.4239 | 0.07071 | 0.7495 |
| Std. Error | 0.2111 | 0.1640 | 12.56 | 10.05 | 0.07881 | 0.1730 | 0.05000 | 0.5300 |
| Lower 95% CI of mean | 1.034 | 1.809 | 61.66 | 48.37 | 1.491 | 1.660 | 8.915 | 1.836 |
| Upper 95% CI of mean | 2.206 | 2.719 | 141.6 | 112.3 | 1.896 | 2.550 | 10.19 | 15.30 |
| P value | **0.0426** | | 0.2341 | | 0.0556 | | 0.2070 | |

**Supplemental Table S17: Overview of potential differences of selected biomarkers between children with VG and OM diet.** Data are taken from the references given. Yellow: No significant difference; red: Significant lower values in VG group compared to OM; green: Significant higher values in VG group compared to OM

| | Reference | Age group [y] | Haemoglobin | Ferritin | 25-OH Vitamin D3 | Vit.B12 | CRP | Triglycerides | HDL-C | LDL-C | Total cholesterol |
|---|---|---|---|---|---|---|---|---|---|---|---|
| 2-10 y | Ambroszkiewicz. J. et al.. 2017 (24) | 4-9 | p=0.394 | **p=0.003** | N/A | | **p=0.011** | N/A | N/A | N/A | N/A |
| | Ambroszkiewicz. J. et al.. 2018 (27) | 5-10 | N/A | N/A | p=0.469 | | N/A | N/A | N/A | N/A | N/A |
| | Ambroszkiewicz et al.. 2023 (29) | 5-9 | N/A | N/A | p=0.869 | | N/A | N/A | N/A | N/A | N/A |
| | Desmond et al.. 2021 (30) | 5-10 | p>0.05[3)] | **p<0.05**[3)] | p>0.05[3)] | p<0.05[5)] | p>0.05[3)] | **p< 0.01**[3)] | **p<0.05**[3)] | p>0.05[3)] | p>0.05[3)] |
| | Alexy et al 2021 (22) | 5-19 | p=0.414[2]) | **p=0.013**[2] | p=0.170[2] | **p=0.0042**[2] | N/A | p=0.057[2] | p=0.595[2] | p=0.070[2] | p=0.120[2] |
| | Elliott et al.. 2022 (31) | 0.5-8 [1)] | N/A | p=0.950 | p=0.140 | | N/A | p=0.550 | p=0.080 | p=0.130 | p=0.390 |
| | Hovinen et al.. 2021 (33) | 1-7 | p=0.71[4)] | p=0.21[4)] | p=0.13[4)] | p>0.05 | p=1.00[4)] | p=0.71[4)] | p=0.78[4)] | p=0.17[4)] | p=0.33[4)] |
| | Svetnicka et al 2022 (38) | 0-18 | | | | **p=0.019** | | | | | |
| | Grant et al.. (2021). (32) | 14-17 [1)] | N/A | N/A | N/A | | p=0.527 | p=0.601 | p=0.830 | **p=0.001** | **p=0.001** |

1) VEs are included in VG group;
2) Data were adjusted adjusted for age. BMI-SDS. socioeconomic status. smoking in the household. physical activity. use of dietary supplements. p-values were adjusted for multiple testing according to the False Discovery Rate (FDR) method.
3) Data were adjusted to diet group. age. sex
4) p-values were calculated using age- and sex-adjusted exact permutation tests with n = 47500 permutations and Benjamini-Hochberg correction for multiple testing
5) without supplementation or fortification

N/A: Not available;
